# Opioid and other drug use and drug-related mortality as indicators of Hepatitis C and Human Immunodeficiency Virus in Oklahoma

**DOI:** 10.1371/journal.pone.0301442

**Published:** 2024-05-09

**Authors:** Aaron M. Wendelboe, Ozair H. Naqvi, Mary Williams, Heather Hollen, Kaitlin McGrew, Peng Li, Terrainia Harris, Ann F. Chou

**Affiliations:** 1 Department of Biostatistics and Epidemiology, Hudson College of Public Health, University of Oklahoma Health Sciences Center, Oklahoma City, OK, United States of America; 2 The Oklahoma State Department of Health, Sexual Health & Harm Reduction Service, Oklahoma City, OK, United States of America; 3 Department of Family and Preventive Medicine, College of Medicine, The University of Oklahoma Health Sciences Center, Oklahoma City, OK, United States of America; Menzies School of Health Research: Charles Darwin University, AUSTRALIA

## Abstract

**Objectives:**

Outbreaks of injection drug use (IDU)-associated infections have become major public health concerns in the era of the opioid epidemic. This study aimed to (1) identify county-level characteristics associated with acute HCV infection and newly diagnosed IDU-associated HIV in Oklahoma and (2) develop a vulnerability index using these metrics.

**Methods:**

This study employs a county-level ecological design to examine those diagnosed with acute or chronic HCV or newly diagnosed IDU-associated HIV. Poisson regression was used to estimate the association between indicators and the number of new infections in each county. Primary outcomes were acute HCV and newly diagnosed IDU-associated HIV. A sensitivity analysis included all HCV (acute and chronic) cases. Three models were run using variations of these outcomes. Stepwise backward Poisson regression predicted new infection rates and 95% confidence intervals for each county from the final multivariable model, which served as the metric for vulnerability scores.

**Results:**

Predictors for HIV-IDU cases and acute HCV cases differed. The percentage of the county population aged 18–24 years with less than a high school education and population density were predictive of new HIV-IDU cases, whereas the percentage of the population that was male, white, Pacific Islander, two or more races, and people aged 18–24 years with less than a high school education were predictors of acute HCV infection. Counties with the highest predicted rates of HIV-IDU tended to be located in central Oklahoma and have higher population density than the counties with the highest predicted rates of acute HCV infection.

**Conclusions:**

There is high variability in county-level factors predictive of new IDU-associated HIV infection and acute HCV infection, suggesting that different public health interventions need to be tailored to these two case populations.

## Introduction

The intersection between injection-drug use (IDU), Hepatitis C virus (HCV) infection, and human immunodeficiency virus (HIV) infection continues to pose challenges to public health [1, 2] as IDU significantly increases the risk of developing a blood-borne infection [2]. Moreover, higher prevalence of IDU may leave communities vulnerable to infectious disease outbreaks, with six major outbreaks of HIV among persons who inject drugs occurring in the United States from 2016–2019 [3]. Prior investigations have shown that over 70% of persons who inject drugs have been diagnosed with HCV, and such activities are linked to nearly 7% of new HIV infections [4, 5]. Epidemiologic commonalities between outbreaks of HCV and HIV associated with IDU include high proportions of young, white, non-Hispanic men who reported use of injection opioids (e.g., heroin and fentanyl) or other stimulants (e.g., methamphetamine and cocaine) [3, 6]. Other common characteristics include homelessness, close-knit social networks, recent incarceration, and use of sex for compensation [1, 2, 3, 6]. Such risks are expected to increase as the opioid crisis continues, with opioid dispensing rates remaining high in certain regions of the United States [7].

Perhaps the most notable event related to IDU, HCV, and HIV infection was the 2014–2015 HIV outbreak in Scott County, Indiana, which was linked to injection use of Oxymorphone [8, 9]. One-hundred-eighty-one cases of new HIV infections were identified, with 92% having a co-infection with HCV and 96% having engaged in IDU [8]. Investigators noted an unexpectedly large network of IDUs and a high prevalence of HCV co-infections [8]. The outbreak highlighted the need to assess the vulnerability of rural communities to HIV outbreaks where IDU and HCV infection are prevalent, as HCV infection can be used as a marker of community risk [8]. In response, the Centers for Disease Control and Prevention (CDC) conducted a county-level vulnerability assessment of communities at risk for rapid HIV and HCV infections among persons who inject drugs [10]. The assessment built on the CDC Social Vulnerability Index, which illustrates which communities are more likely to be affected during a disaster [11]. Counties identified as high-risk were rural and with lower socioeconomic status [11].

Oklahoma continues to face substantial morbidity due to HCV and HIV, with 0.5 per 100,000 [12] reported cases of acute HCV infection in 2020 and an estimated 9.3 per 100,000 [13] new cases of HIV infection among persons aged greater than 13 years from 2015–2019. In 2020, Oklahoma ranked eighth in the United States in opioid dispensing rate, with 59.8 per 100,000 [7]. Following the national vulnerability assessment, jurisdictions began using local data to build on public health emergency preparedness efforts involving HCV, HIV, and IDU [14, 15]. It should be noted that two of the top 220 highest-risk counties were located in Oklahoma [10]. However, a comprehensive risk assessment of Oklahoma counties is absent, which hampers the ability of health departments to respond in the event of outbreaks. The goal of this study was to identify demographic and community-level characteristics that are associated with rates of HIV and HCV infection in Oklahoma. We further aimed to develop a vulnerability index using these metrics as a tool to provide local public health jurisdictions with additional insight into identifying communities vulnerable to the initiation of IDU-associated HIV/HCV outbreaks and tailor targeted interventions to reduce morbidity and mortality related to these conditions.

## Methods

### Study design

The study employed an epidemiological ecological design [16] to evaluate the association between population-level characteristics and acute HCV infection and newly diagnosed HIV infection from IDU (HIV-IDU). County-level aggregate, cross-sectional data were used to estimate the vulnerability of each Oklahoma county to experience an outbreak from IDU infections. Ecological studies are used for generating hypotheses by measuring correlations between independent and dependent variables that apply to groups of people. The interpretation of results from an ecological study should not be applied to individuals. The University of Oklahoma Health Sciences Center Institutional Review Board determined this activity was not human subjects research (IRB#15469).

### Data sources

This study included datasets from the American Community Survey/U.S. Census Bureau, CDC, National Center for Health Statistics, Sexual Health and Harm Reduction Service (SHHRS) of the Oklahoma State Department of Health (OSDH), the Oklahoma State Bureau of Investigation, and the Oklahoma State Bureau of Narcotics and Dangerous Drugs Control. Surveillance data were obtained from SHHRS for HCV infection and HIV infection diagnosed during 2015–2017. Surveillance data are published by OSDH two years after the year they are reported (e.g., 2017 data were available in 2019). Only newly diagnosed HIV cases whose primary mode of transmission was identified as either “IDU” or “IDU and male-to-male sexual contact” were eligible for inclusion. Acute HCV cases, classified as either confirmed or probable, were also included. Chronic HCV cases were eligible for inclusion in the sensitivity analyses described below. Of note, data for 2019 onward were significantly delayed due to the COVID-19 pandemic, as the OSDH prioritized activities related to pandemic response.

Selection of variables entered into the model was informed by previously published vulnerability analyses [10, 14, 17], and supplemented with the authors’ experience working in public health in Oklahoma. They include age (15–24, 25–44, 45–59, and ≥60 years), sex (male and female), ethnicity/race (Hispanic [any] and non-Hispanic: white, black, Native American, Asian, Pacific Islander, and two or more races), poverty (percent at or below the federal poverty level), education level (percent of the county aged 18–24 years with less than a high school education), population density (number of people living per square mile), health insurance status (percent uninsured), adult drug-related crime rate (number of crimes per county population), opioid-related death rate (number of deaths per county population), and opioid prescribing rates. The population in 2016, the median year of the analysis, was used for the population denominator, age, sex, race, poverty, insurance status, education level, and population density. Collinearity of variables was assessed by using Pearson’s correlation. Overdispersion was assessed by evaluating the dispersion chi-square statistic. In addition, similar results were obtained when using a negative binomial distribution.

### Statistical analysis

County-level characteristics were categorized as either the percentage of the county with a given characteristic or the rate (e.g., the number of events divided by the county population) for that county. As a proxy measure for IDU, the primary outcomes of interest were (1) all reported new HIV cases with evidence of IDU, and (2) all reported acute HCV cases. A sensitivity analysis was conducted to examine all HCV (acute and chronic) cases. Cases with missing county of residence were excluded from the analyses. Poisson regression was used to estimate the association between covariates and the number of new infections in each county. A backward stepwise model-building approach was implemented. A crude analysis of each covariate and the outcome was performed; variables with an *a priori* p≤ 0.20 were considered for the next step in the full model. The percentage of the population by age group variables were correlated with one another, as were nearly half of the race categories; therefore, the variable with the lowest p-value was selected for use in the full model. Projected new infection rates and 95% confidence intervals (CI) were generated for each county from the final multivariable model. Specifically, estimated log-counts of new infections were derived in SAS v.9.4 (Cary, NC) with PROC GENMOD, converted to log-rates, and exponentiated in PROC PLM for final interpretation. These predicted new infection rates served as our metric for vulnerability scores, and counties were directly ranked from highest to lowest vulnerability based on descending new infection rates.

## Results

Of the 77 counties in Oklahoma, 76 had completed ecological data and reported opioid prescribing rates, except Beaver County, which did not report any opioid prescribing data. Of the 76 counties, 24 (31.2%) had ≥ 1new HIV-IDU infection and 48 (62.3%) counties had ≥ 1 acute HCV infection during 2015–2017. In all, there were 111 patients with a new diagnosis of HIV with IDU as a risk factor, 266 with acute HCV, and 8,483 patients with chronic HCV. Twenty counties (26%) reported more than 100 cases of HCV (acute and chronic). These included Beckham, Bryan, Canadian, Cherokee, Cleveland, Comanche, Creek, La Flore, Mayes, Muskogee, Oklahoma, Okmulgee, Osage, Payne, Pittsburg, Pottawatomie, Rogers, Sequoyah, Tulsa, and Wagoner. The three most populated counties in the state reported the highest number of cases: Cleveland (n = 854), Oklahoma (n = 1,368), and Tulsa (n = 1,478).

When assessing possible collinearity, each age group variable exhibited significant intercorrelation (p<0.001) with absolute values for correlation coefficients ranging from 0.39–0.83. The correlation among race/ethnicity variables was less consistent, where percent white, Native American, and two-or-more races were largely intercorrelated, while black, Asian, Pacific Islander, and Hispanic tended to not be intercorrelated.

Most of the predictors for new IDU-associated HIV infection differed from acute HCV infection; the direction of association (positive vs. negative) as indicated by the β-estimates for 15 of the 19 predictors were opposite between the two infections (Table 1). HIV-IDU was positively associated with percent of the population aged 25–44 years, percent black race, percent Asian race, counties with a high opioid prescribing rate, and high population density. HIV-IDU was negatively associated with percent of the population aged 45–59 years and 60+ years, percent white race, Native American race, and being of two or more races. Acute HCV infection was positively associated with percent of the population that is male, percent aged 45–59 years and 60+ years, percent Native American, percent two or more races, having a high prescription opioid-related death rate, higher precent living below the poverty line, percent uninsured, and percent with less than a high school education in the age group 18–24 years. HCV infection was negatively associated with percent of the county population aged 25–44 years, percent black race, Asian race, Pacific Islander race, and population density. It is worth noting that the magnitude of association was much larger for the variable prescription opioid-related death rate, with the number of infections, compared to all other variables.

**Table 1:** County-level characteristic estimates associated with the number of infections of (1) new IDU-associated HIV infections and (2) acute HCV infections, based on crude Poisson regression models, with p-value established at 0.05 for statistical significance.

Indicator	HIV-IDU			Acute HCV		
	Estimate	SE	p-value	Estimate	SE	p-value
Percent male	-0.204	0.106	0.054	**0.168**	**0.042**	**<0.0001**
Percent aged 15–24 years	0.013	0.027	0.636	-0.030	0.021	0.158
Percent aged 25–44 years	**0.166**	**0.035**	**<0.0001**	**-0.0836**	**0.0188**	**<0.0001**
Percent aged 45–59 years	**-0.192**	**0.062**	**0.002**	**0.179**	**0.046**	**<0.0001**
Percent aged 60+ years	**-0.141**	**0.037**	**0.0001**	**0.077**	**0.016**	**<0.0001**
Percent white	**-4.209**	**-6.39**	**0.0002**	1.253	0.692	0.070
Percent black	**9.520**	**1.770**	**<0.0001**	**-5.922**	**1.21**	**<0.0001**
Percent Native American	**-7.887**	**2.216**	**0.0004**	**4.400**	**0.668**	**<0.0001**
Percent Asian	**34.004**	**7.075**	**<0.0001**	**-21.685**	**4.300**	**<0.0001**
Percent Pacific Islander	18.480	21.603	0.392	**-64.263**	**32.641**	**0.049**
Percent Hispanic	-1.332	1.946	0.494	-0.418	1.165	0.720
Percent two or more races	**-13.638**	**6.480**	**0.035**	**24.993**	**3.543**	**<0.0001**
Prescription opioid-related death rate	556.834	744.886	0.455	**1474.303**	**453.877**	**0.001**
Opioid prescribing rate	**0.010**	**0.004**	**0.0068**	0.000	0.0019	0.981
Percent below poverty line	0.008	0.030	0.800	**0.042**	**0.019**	**0.024**
Drug crime rate	-10.700	53.48	0.842	27.824	31.703	0.380
Percent uninsured	-0.030	0.033	0.355	**0.059**	**0.018**	**0.001**
Percent with less than high school education in age group 18–24	-0.032	0.018	0.080	**0.042**	**0.012**	**0.0004**
Population Density	**0.001**	**0.0002**	**<0.0001**	**-0.001**	**0.000**	**<0.0001**

When applying the stepwise backward selection process to identify the most useful factors, the parsimonious HIV-IDU model differed from acute HCV (Table 2). New IDU-associated HIV infection was most strongly associated with higher population density and weakly negatively associated with percent of the population aged 18–24 years with less than a high school education. In contrast, final predictors for acute HCV infection included percent of the county that was male, white, two or more races, and percent of the population aged 18–24 years with less than a high school education. The percent of the population that was Pacific Islander was weakly negatively associated with acute HCV.

**Table 2:** List of variables selected via stepwise backward selection for each model to assess relationships between county-level characteristics and opioid, other drug use, and drug-related mortality as indicators of newly diagnosed cases of IDU-associated HIV infection and acute HCV infection.

New HIV-IDU	Acute HCV
• Percent of aged 18–24 years with less than High School education (β = -0.038, p = 0.097) • Population density (β = 0.0013, p< 0.001)	**•** Percent male (β = 0.135, p = 0.003) **•** Percent white (β = 1.407, p = 0.066) **•** Percent Pacific Islander (β = -55.00, p = 0.120) **•** Percent two or more races (β = 24.32, p < 0.001) **•** Percent of aged 18–24 years with less than High School education (β = 0.024, p = 0.046)

**Fig 1:**
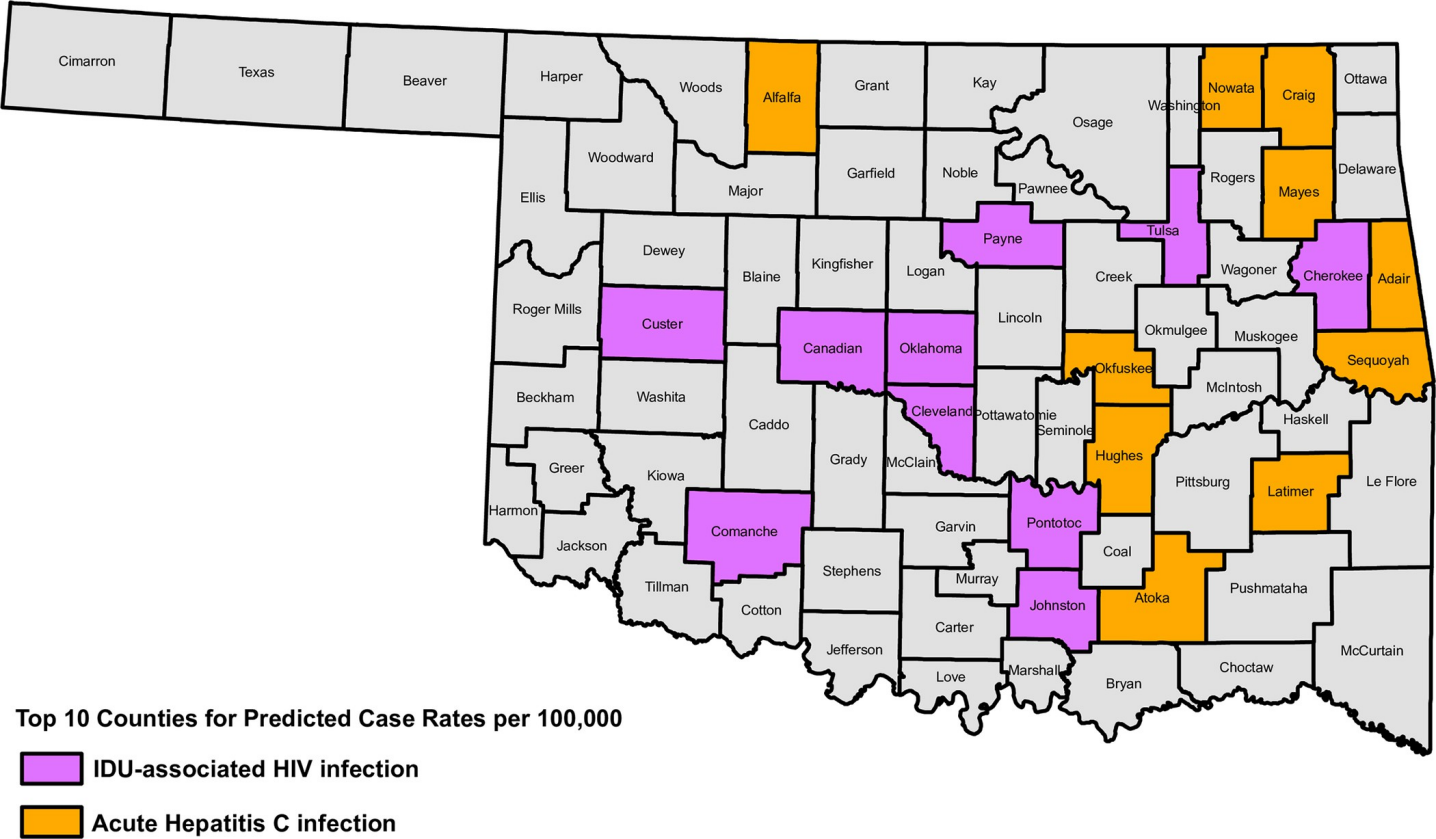
Map of counties in Oklahoma that have the ten highest predicted rates of new IDU-associated HIV infections (purple) and acute HCV infections (orange).

Predicted rates (and 95% confidence intervals) of new IDU-associated HIV infection and acute HCV infection are shown in Table 3. Counties with the highest predicted rates for HIV-IDU tended to be in the center of the state, while counties with the highest predicted rates for acute HCV infection tended to be in the eastern portion of the state (Fig 1).

**Table 3:** Predicted rates of new IDU-associated HIV infection and acute HCV by county^a^.

COUNTY	New HIV-IDU			Acute HCV		
	Rate /100,000	Lower CI	Upper CI	Rate /100,000	Lower CI	Upper CI
Adair County	1.0289	0.5983	1.7694	16.8761	10.8193	26.3237
Alfalfa County	1.3101	0.9066	1.8933	15.8926	6.2406	40.4724
Atoka County	1.2458	0.8455	1.8358	15.2839	11.3406	20.5984
Beckham County	0.9316	0.5004	1.7343	6.7617	4.2073	10.8669
Blaine County	1.248	0.8479	1.8368	10.8574	6.7253	17.5281
Bryan County	1.4952	1.0596	2.11	8.1043	6.568	10
Caddo County	1.0304	0.6086	1.7445	8.9015	6.3204	12.5365
Canadian County	1.7473	1.2632	2.417	4.7532	3.7602	6.0084
Carter County	0.933	0.4849	1.7953	8.5153	6.2789	11.5481
Cherokee County	1.7497	1.1943	2.5634	9.8824	6.7601	14.4468
Choctaw County	1.1321	0.7234	1.7718	7.535	6.1633	9.2119
Cimarron County	1.188	0.7901	1.7865	3.6952	2.7055	5.0469
Cleveland County	3.4903	2.2421	5.4333	5.2325	3.9145	6.9944
Coal County	1.0187	0.6016	1.725	13.9118	10.9668	17.6477
Comanche County	1.9627	1.3207	2.9166	4.8994	3.087	7.7758
Cotton County	0.9422	0.5191	1.7103	7.1452	5.3142	9.607
Craig County	1.1759	0.7711	1.7931	17.0341	13.1954	21.9894
Creek County	1.2756	0.8582	1.8959	9.8112	7.9742	12.0713
Custer County	1.8386	1.1683	2.8934	3.224	2.3722	4.3817
Delaware County	1.2377	0.8238	1.8594	11.2977	9.5625	13.3479
Dewey County	1.0691	0.6605	1.7303	6.4725	4.6728	8.9654
Ellis County	0.9523	0.5331	1.7012	3.9419	2.4124	6.4413
Garfield County	1.4701	1.0453	2.0674	0.9391	0.1503	5.8687
Garvin County	1.0984	0.6788	1.7774	7.408	5.893	9.3125
Grady County	1.2761	0.8681	1.8759	6.4374	4.9676	8.3421
Grant County	1.5384	1.056	2.2411	4.4149	3.0004	6.4962
Greer County	0.9936	0.5748	1.7174	13.9558	6.2907	30.9605
Harmon County	0.7489	0.3284	1.708	5.2346	3.2604	8.4041
Harper County	0.7275	0.3099	1.7076	3.9995	2.3176	6.9019
Haskell County	1.0289	0.6076	1.7426	12.404	10.1149	15.2112
Hughes County	0.9444	0.5176	1.7231	18.327	11.9967	27.9975
Jackson County	1.5021	1.0569	2.1349	2.8639	2.1528	3.81
Jefferson County	0.8174	0.3915	1.7066	8.3658	5.7123	12.2519
Johnston County	1.7455	1.1362	2.6815	9.58	7.0987	12.9287
Kay County	1.0882	0.6588	1.7976	6.5918	5.0701	8.5704
Kingfisher County	1.1107	0.7011	1.7597	4.6029	3.3622	6.3016
Kiowa County	1.2853	0.8842	1.8684	4.3863	3.4444	5.5857
Latimer County	1.0963	0.6857	1.7527	15.7783	12.6514	19.678
Le Flore County	1.1455	0.7328	1.7908	8.3216	7.0431	9.8321
Lincoln County	1.1875	0.7772	1.8143	7.7437	5.89	10.1809
Logan County	1.4683	1.0438	2.0656	5.0341	3.9852	6.3591
Love County	1.143	0.7359	1.7752	6.1411	5.0045	7.5357
Major County	0.7206	0.3023	1.7177	5.9433	3.4422	10.2617
Marshall County	1.0136	0.5797	1.7722	7.8763	6.2415	9.9393
Mayes County	1.0769	0.6391	1.8145	18.488	14.2652	23.9607
McClain County	1.551	1.1026	2.1819	6.8368	5.4269	8.6129
McCurtain County	0.9307	0.5028	1.7229	6.4929	3.8219	11.0308
McIntosh County	1.2422	0.8371	1.8433	10.9978	9.3416	12.9475
Murray County	1.2046	0.7972	1.82	9.0074	7.589	10.6909
Muskogee County	1.3239	0.9054	1.9357	10.0853	8.1367	12.5004
Noble County	1.148	0.7424	1.7751	5.9726	4.4957	7.9348
Nowata County	1.1434	0.7363	1.7758	17.2558	13.3231	22.3492
Okfuskee County	0.8914	0.4607	1.7248	16.7684	10.8086	26.0146
Oklahoma County	4.9948	3.9815	6.266	3.9123	3.0225	5.0641
Okmulgee County	1.4321	1.015	2.0205	12.3757	9.9252	15.4313
Osage County	1.2329	0.8308	1.8296	10.4484	8.9416	12.2092
Ottawa County	1.4291	1.0135	2.0152	6.2798	3.5563	11.0888
Pawnee County	1.1905	0.7833	1.8095	8.895	7.297	10.8428
Payne County	2.4085	1.3513	4.293	6.0053	4.1007	8.7946
Pittsburg County	1.0952	0.6749	1.7774	14.3022	11.5896	17.6497
Pontotoc County	1.706	1.1678	2.4922	8.1833	6.4053	10.4549
Pottawatomie County	1.4294	1.0134	2.0161	6.5108	5.0603	8.3769
Pushmataha County	0.8234	0.3975	1.7058	8.5916	6.1857	11.9332
Roger Mills County	1.0155	0.6022	1.7125	3.9322	2.5752	6.0041
Rogers County	1.6689	1.2169	2.2889	12.2648	9.6342	15.6137
Seminole County	1.0536	0.6254	1.7748	9.3597	7.6473	11.4554
Sequoyah County	1.0566	0.6163	1.8115	18.7833	14.2821	24.7031
Stephens County	1.2213	0.8077	1.8467	5.7999	4.3406	7.7497
Texas County	1.1054	0.6982	1.7501	2.8809	1.5075	5.5056
Tillman County	0.9928	0.5743	1.7164	4.7685	3.2208	7.0599
Tulsa County	4.8667	3.7917	6.2465	5.3556	4.5114	6.3577
Wagoner County	1.3316	0.8939	1.9837	11.0133	9.2218	13.1529
Washington County	1.6499	1.2009	2.2667	6.8296	5.5353	8.4265
Washita County	0.846	0.4183	1.7108	5.9802	3.8339	9.3278
Woods County	1.4857	1.0317	2.1396	6.5242	4.1076	10.3624
Woodward County	1.3419	0.9346	1.9269	5.1601	3.4432	7.7331

Beaver County is excluded due to missing opioid prescribing rate data.

Results from the sensitivity analysis for all HCV (acute and chronic) infections are included in the Supplementary file. The number of all HCV infections was more than 30 times greater; the univariate and adjusted models for all HCV infection had more predictors that were significantly associated, but the counties with the highest predicted rates of all HCV infections tended to overlap with the counties that had the highest predicted rates of acute HCV infection.

## Discussion

This report presents the landscape of IDU-related infections, focusing on HCV and HIV, across the state of Oklahoma, computing predicted rates of infection for 76 of 77 counties. The key finding to highlight is that county-level characteristics differed greatly between new IDU-associated HIV infection and acute HCV infection. Thus, while both might be a proxy for injection drug-use and that CDC and health departments tend to combine those cases for reporting due to small HIV numbers, it is not appropriate to aggregate them into a single category for predictive modeling purposes. Given these differences, it is more difficult for the health department to identify counties at the highest risk of an outbreak of infections related to injection drug use. Further, as discussed below, Oklahoma’s county-level predictors differed from those in other states, demonstrating the importance of not overgeneralizing the characteristics of vulnerable populations.

Higher rates of HIV-IDU were associated with higher population density and more individuals aged 18–24 years with a high school education. In contrast, higher rates of acute HCV were associated with lower population density, fewer people aged 18–24 years with a high school education, and counties with higher percentages of people who are male, white race, or two or more races.

This vulnerability assessment was conducted in the context of approximately 40 other states, each undertaking its own analysis. Although each state had flexibility in designing the methods of their analyses, regular conference calls were hosted by CDC and the Council of State and Territorial Epidemiologists (CSTE) to provide technical assistance and facilitate comparability across states. When comparing the results in the current report to those published and ongoing, there was a difference in the indicator variables identified as having significant associations. Among those analyses assessing the vulnerability of Oklahoma Counties, [10, 14, 17] there is an observed difference in the most vulnerable counties. Specifically, Van Handel et al. identified Jefferson and Cimarron Counties as high risk during their national assessment using 2012–2013 data. These differences are expected because each of these analyses (1) was conducted during a different timeframe, (2) used different outcome measures, and (3) had access to different independent variables. Due to these differences, we caution against relying too heavily on the statistical significance of any one independent variable or the numeric value of predicted rates of infection. Instead, results from this report will be most helpful in identifying potential factors that can be addressed by public health officials, the medical community, and the general public.

In contrast to national reports, which focused on HIV infection, of having a higher representation of white, young and rural population demonstrating an association with increased vulnerability [10], our analyses showed that counties with higher percentages of white race, those of two or more races, and rural population were associated with acute HCV infection, but not HIV-IDU. When comparing to the findings by Rickles et al. in Tennessee, we found that high rates of prescribing buprenorphine was a predictive factor in terms of a large β estimate; however, it did not remain statistically significant in adjusted models [14].

**Fig 2:**
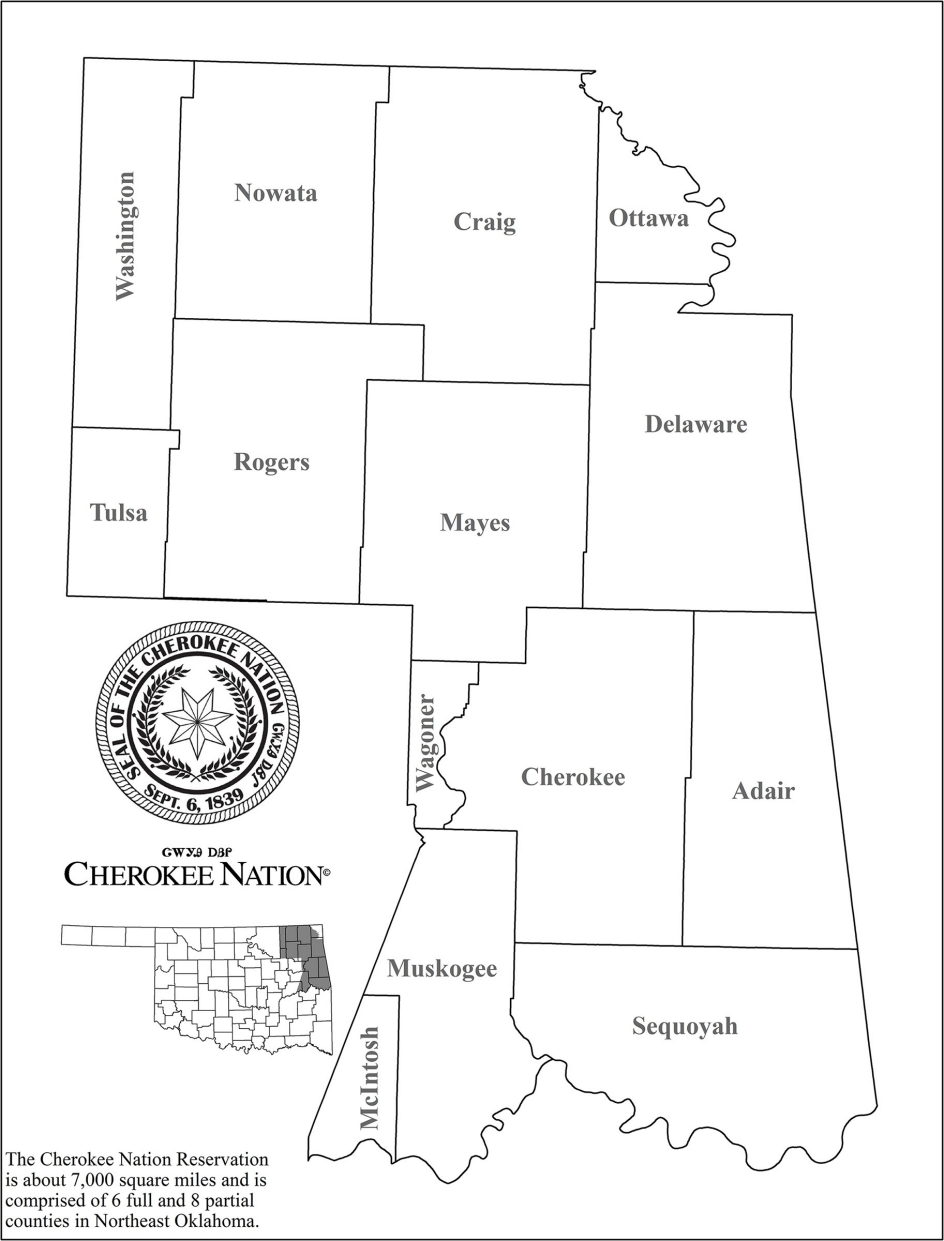
Overlap of the Cherokee Nation jurisdiction and counties in Oklahoma, many of which ranked high on the acute HCV and IDU-associated new HIV infection vulnerability assessment. This map was provided to the authors under a CC BY license, with permission from Cherokee Nation, original copyright 2023.

There are a number of limitations to note for this study. First, there may be possible diagnosis or surveillance bias in the 14 counties within the Cherokee Nation jurisdictional area (Fig 2). The Cherokee Nation developed and implemented an extensive HCV elimination program in 2015. This program is ongoing with screening of all those accessing the Cherokee Nation Health System aged 20–69 years, which may result in more cases identified. Subsequently, these counties may be more prepared to address the public health challenges associated with IDU. Next, backward stepwise selection may have resulted in inflated regression coefficient and R^2^ values, as well as narrowed confidence intervals and deflated p-values. While Poisson models are less prone to overfitting when sample sizes are relatively large, measures to prevent overfitting, such as regularization or cross-validation, were not explicitly implemented here, which may limit the generalizability of our findings.

In addition, counties with the lowest reported infection rates may be associated with external factors and not a result of low infection rates. The use of HCV and IDU-associated HIV infection status as a proxy for using intravenous drugs likely underestimated the true burden of intravenous drug use in the community. It is possible we double-counted some individuals who were co-infected with HIV and HCV. Personal identifying information was not available to determine the uniqueness of each individual in the dataset. However, differences in predictive characteristics between the new IDU-associated HIV cases and the acute HCV cases suggests that any overlap would bias any estimates towards the null. Counties without a single case may potentially introduce inflated variance in the Poisson regression model. We addressed this limitation in two ways. We first compared results across models, as zero counts may have impacted the primary model (of acute cases only), but not the models including chronic HCV cases. The results were comparable across models. We then requested technical assistance from the CDC/CSTE who helped us arrive at our analytic plan. Second, this vulnerability analysis is an ecological study and includes data from multiple publicly available sources. While the variables from these sources help identify important associations, they may not be designed to predict rates of infections. HCV and IDU-associated HIV infection status was used as a proxy for IDU. The assumption that HCV has been contracted via IDU may be flawed, as only 73.6% of the acute HCV cases diagnosed in Oklahoma during 2015–2017 identified IDU as a mode of transmission (internal surveillance data). However, without a measure that captures the mode of acute HCV transmission, the analysis can only rely on the use of this proxy.

## Conclusions

This report serves to inform and educate medical providers and public health departments by identifying counties vulnerable to IDU-associated outbreaks of HIV or acute HCV. We identified county-level indicators that are associated with an increased number of infections for targeted prevention and intervention efforts.

The American Indian/Native American communities in northeastern Oklahoma may be at increased risk for poor outcomes associated with IDU. However, they may have a higher awareness of HCV and HIV infections in their respective communities. Those counties with higher tribal populations also have more robust HCV screening in place and may consequently identify more cases.

Communities that previously experienced outbreaks related to IDU have reduced the risk of disease transmission by instituting safe needle exchange programs [8]. Continued collaborative efforts are needed to engage stakeholders in identifying strategies for prevention that are tailored to the characteristics of the community and the needs of its members in reducing IDU and preventing IDU-associated outbreaks. Approaching intravenous drug misuse as a chronic illness and, therefore applying behavioral and medication-assisted treatments that are evidence-based is recommended [18].
